# Metal additive manufacturing and possible clinical markers for the monitoring of exposure-related health effects

**DOI:** 10.1371/journal.pone.0248601

**Published:** 2021-03-18

**Authors:** Stefan A. Ljunggren, Liam J. Ward, Pål Graff, Anders Persson, Malin Leijon Lind, Helen Karlsson

**Affiliations:** 1 Department of Health, Medicine and Caring Sciences, Occupational and Environmental Medicine Center in Linköping, Linköping University, Linköping, Sweden; 2 Department of Clinical Sciences, Intervention and Technology, Karolinska Institutet, Stockholm, Sweden; 3 National Institute of Occupational Health, Oslo, Norway; 4 Siemens Energy AB, Finspång, Sweden; Government College University Faisalabad, PAKISTAN

## Abstract

Additive manufacturing (AM) includes a series of techniques used to create products, in several different materials, such as metal, polymer or ceramics, with digital models. The main advantage of AM is that it allows the creation of complex structures, but AM promises several additional advantages including the possibility to manufacture on demand or replacing smaller worn parts by directly building on an existing piece. Therefore, the interest for and establishment of AM is rapidly expanding, which is positive, however it is important to be aware that new techniques may also result in new challenges regarding health and safety issues. Metals in blood and possible clinical effects due to metal exposure were investigated in AM operators at one of the first serial producing AM facilities in the world during two consecutive years with implementation of preventive measures in-between. As comparison, welders and office workers as control group were investigated. Health investigations comprised of surveys, lung function tests, antioxidant activity and vascular inflammation as well as renal- and hepatic function analysis. AM operators had significantly reduced nickel levels in blood (10.8 vs 6.2 nmol/L) as well as improved lung function (80 vs 92% of predicted) from year 1 to year 2. This is in line with previously published results displaying reduced exposure. Blood cobalt and nickel levels correlated with previously reported urinary levels, while blood chromium did not. Multivariate modelling showed that blood cobalt, antioxidant/inflammatory marker serum amyloid A1/serum paraoxonase/arylesterase 1 activity and the hepatic markers aspartate transaminase, alanine transaminase, and alkaline phosphatase were higher in AM operators compared to controls. The study show that the selected clinical analyses could function as a complement to metal analyses in biological fluids when investigating exposure-related health effects in AM operators. However, validation in larger cohorts is necessary before more definite conclusions could be drawn.

## Introduction

Additive manufacturing (AM) is represented by a series of techniques used to create products, with several different materials such as metals, polymers or ceramics, from the bottom-up using digital models [1]. The main advantage of AM is that it allows the creation of complex structures, which means that the technology is an excellent complement to traditional manufacturing techniques [2]. AM also promises several additional advantages over traditional manufacturing methods, including the possibility to manufacture on demand and thereby reducing the need to keep components in stock or replacing smaller worn parts by directly building on an existing piece. Therefore, the interest for and establishment of AM is today rapidly expanding but it is important to be aware that new techniques may also result in new challenges regarding the environment, and health and safety.

Access to guidelines for exposure related health effects in AM environments is still limited. However, regarding polymer AM, there are available scientific reports describing health effects such as contact dermatitis, asthma and allergic rhinitis [3–5]. In contrast, an experimental exposure study of volunteers to polymer materials for 1 h showed no acute effect on inflammatory markers in nasal secretions or urine but increased exhaled nitric oxide [6]. For metal AM, there are still no reports addressing health effects in operators. Since metal AM involve both ultrafine and fine metal particles, and in general similar elements as can be found in metal production environments, it could be expected that AM operators have similar exposure related health risks as other metalworkers [7,8]. For example, lung complications have been found in hard metalworkers due to cobalt, hexavalent chromium, and nickel exposure [9–11], which may be relevant since cobalt, nickel, and chromium (but not hexavalent) are common components in AM powders. Also, lung function and cardiovascular disease (CVD) risk among welders have been extensively studied [12–15]. Moreover, the risk of negative effects on renal function as result of chromium exposure [16] and hepatotoxic effects as result of chromium exposures have been suggested [17].

To monitor the possible health effects in the AM environment, traditional methods including spirometry as well as measuring metals in blood may be useful for an initial assessment. These methods may also be informative when comparing the AM environment with other occupational settings that have a metal exposure. Due to the limited data on possible effects of metal AM, a broad range of markers for effects in various tissues are needed, as a first step, to try to identify organs that are affected. The liver represents a possible target organ for metals exposure-related disease [18], and commonly clinically used liver markers in blood such as aspartate aminotransferase (ASAT), alanine aminotransferase (ALAT) and alkaline phosphatase (ALP) are used for monitoring. Another organ of interest is the kidney, the urine marker of α1-microglobulin that could identify renal tubular damage [19] could be of interest. For the cardiovascular system there exist many different blood metrics including lipoproteins, apolipoprotein A-I (apo A-I) and apolipoprotein B (apo B) are routinely used in the healthcare. Besides its protective effects against cardiovascular disease due to the reverse cholesterol transport, apo A-I situated on high-density lipoprotein (HDL) has important effects in immune defense [20,21]. Furthermore, apo A-I is present on most studied nanoparticles in plasma [22,23]. Therefore, apo A-I may be involved in nanoparticles clearance via the scavenger receptor B-I on hepatocytes, followed by distribution via bile to feces similar to excess cholesterol and endotoxins, making it interesting for metal occupational exposure. Besides apo A-I, HDL also contains other proteins such as the acute phase protein serum amyloid A1 (SAA1) and the antioxidant protein serum paraoxonase/arylesterase 1 (PON1). These both are highly involved in CVD risk [24] and may be affected by the presence of nanoparticles making them of interest for occupational metal exposures studies.

Our group has previously published occupational exposure data from one of the world’s first metal AM serial production plants [7,8], where a common AM technique has been studied; selective laser melting (SLM) also called powder bed fusion-laser beam (PBF-LB). These measurements included dust, metal, and particle levels in air as well as exposure markers in AM operators in the form of urine and dermal/skin exposure to metals. Besides traditional gravimetric analyses, the distribution of fine and ultrafine particles in surrounding air was mapped and compared to welding environments and occupational metal exposure was confirmed in both welding and AM environments [8]. In the present study, we aim to investigate various parameters and markers for possible health effects, in the same individuals as described above, to further increase the understanding of possible effects of the occupational exposure of metal AM. Furthermore, such analyses for specified parameters/markers can be evaluated for future use for AM operators.

## Methods

### Participants

All participants described in Table 1, were part of a previously published study investigating emissions of airborne particles in AM environments [8]. The participants were recruited from the available workforce at the company at the time of the study. The AM operators represented the workforce in this rapidly expanding unit. During both years of sampling, the operators worked mainly with an alloy powder containing 47% nickel, 22% chromium, 18% iron, 9% molybdenum, 1.5% cobalt, and other metals (below 1%, nominal composition according to the producer) [8]. Controls were recruited from an office in the same building as the AM production. Welders were also recruited from the same company but working in a different building with more traditional manufacturing, however, with similar components as the AM operators. The welders worked with both metal active gas (MAG) welding in unalloyed steel as well as tungsten inert gas (TIG) welding in stainless steel. Recruitment was based on that all available employees of the AM center, office and welding unit who were invited to participate and those willing were included. All samples were collected within 2 weeks during spring both year 1 and year 2.

**Table 1:** Sex and age distribution in controls, AM operators and welders.

		Control	AM-operators	Welders
**Year 1**	Sex (M/F)	6/5 #	6/1	11/0 #
	Age (mean ± SD)	40 ± 8	40 ± 10	40 ± 13
	BMI (mean ± SD)	25,4 ± 2,4 *	27,0 ± 3,8	27,8 ± 1,9 *
	Years worked in current occupational setting (no of participants)			
	<2	4	7	4
	2–5	2	0	4
	>5	5	0	3
**Year 2**	Sex (M/F)	6/3	8/4	
	Age (mean ± SD)	40 ± 11	37 ± 9	
	BMI (mean ± SD)	24.5 ± 2.8	26.9 ± 3.2	
	Years worked in current occupational setting (no of participants)			
	<2	4	9	
	2–5	2	3	
	>5	3	0	

AM–additive manufacturing.

* p<0.05 Welders vs control T-test

# p<0.05 Welders vs control Chi-square.

Blood and urine from AM operators and controls were sampled at a Monday and Friday during one workweek during two consecutive years. Welders were only sampled the first year. For clinical analyses, blood was collected in lithium heparin tubes, centrifuged at 1200 G for 12 min, before plasma was transferred to new tubes. Whole blood for metal analysis was collected in sodium heparin tubes similar to a previously published study [25]. All samples were directly frozen in -20°C and kept so until analysis.

The study was approved by the Regional Ethics Board in Linköping with approval number 2016/112-31. A signed written informed consent was collected from all participants, which are stored at the Occupational and Environmental Medicine in Linköping.

### Surveys

To evaluate the working environment and work-related symptoms among the employees (AM operators, welders, and office controls), a standardized work environment questionnaire (MM040NA [26,27] was used. In total, 16 AM operators (7 operators in year 1, and 9 operators in year 2), 10 welders and 14 controls (11 controls in year 1, and 3 controls in year 2) completed the questionnaires at their first enrollment. The survey instrument is available in supplementary material (Supplemental S1 File).

### Metal analysis in blood

Metal analysis was performed following a standard protocol for assessment of occupational metal exposure at the Department of Occupational and Environmental Medicine, Linköping University. In short, blood was diluted 15 times using 1% HNO_3_ and analysed using inductively coupled plasma mass spectrometer (ICP-MS, iCAP™ RQ: Thermo Fisher Scientific, Waltham, MA, USA) operated with Ni skimmer cone with 3.5 mm skimmer cone insert (High Matrix), MicroMist 0.4 mL nebulizer, Cyclonic quartz spray chamber and ESI 4DX auto sampler. The instrument settings and parameters were as followed: RF power set on 1549 W, Argon gas flow rate at 14 L/min for plasma and 1 L/min for carrier gas. The uptake speed was 200 μL/min and uptake time 30. The S/C temperature was 2.68°C and dwell time was 0.01, 0.05 or 0.1 s depending on analyte. The conditions of the instrument were optimized using a tuning solution before analyses. Samples were compared to a standard containing metals of interest with concentrations ranging from 0,01–1 μg/L. Seronorm™ Certified Reference Materials, Seronorm Trace Elements Blood levels L-1 and L-2 (low and high levels, respectively) were obtained from Sero (Billingstad, Norway) and were used for analytical quality control. The Reference Materials were reconstituted according to the recommendation of the supplier by adding pure water and were analysed between every tenth samples during the blood analytical runs. For external quality control the laboratory was certified through participating in an inter-laboratory quality program INSTAND e.V. (Düsseldorf, Germany) for blood every 6 month.

### Clinical analyses in blood and urine

Hepatic function was investigated by analysis of ALAT, ASAT and ALP in plasma. Cardiovascular status was investigated by analysis of apo A-I and apo B in plasma and renal function was investigated by α1-microglobulin analysis in urine. All clinical analyses were performed at the Clinical Chemistry Laboratory, Linköping University Hospital, Sweden.

### Serum paraoxonase/arylesterase 1 (PON1) activity

Serum Paraoxonase/Arylesterase 1 (PON1) activity was measured in plasma as previously described [28]. Briefly, lithium heparin plasma was diluted 1:80 with a salt buffer (20 mM Tris–HCl and 1.0 mM CaCl_2_) and a triplicate of 20 μl diluted plasma were added to the wells in a UV-transparent 96-well plate (Sigma–Aldrich). The volume 200 μl of phenyl acetate solution, containing 3.26 mM phenyl acetate in salt buffer, was added to each well and the absorbance of produced phenol was measured at 270 nm in a Clariostar plate reader (BMG Labtechnologies, Offenburg, Germany). The initial period when the reaction was linear was used for calculation of activity, expressed as U/ml, using an extinction coefficient of phenol of 1310 M^−1^cm^−1^.

### Serum amyloid A (SAA1) enzyme-linked immunosorbent assay

To investigate the acute phase response by SAA1, plasma SAA1 levels were measured by an enzyme-linked immunosorbent assay (DY3019-05; R&D systems, Minneapolis, MN) as previously described [29]. In short, citrate plasma was added to the plate and incubated for 2 hours at room temperature. Following wash, a detection antibody was added and incubated for 2 hours. The plate was washed, and streptavidin-horseradish peroxidase was added followed by incubation for 20 minutes. The plate was then washed a final time before a substrate solution was added before 20 minutes incubation. At the end of the incubation, stop solution was added and absorbance was measured at 450 nm with correction at 570 nm using a Clariostar plate reader (BMG Labtechnologies, Offenburg, Germany).

### Spirometry

Lung function of participants was investigated using spirometry. During year 1 AM operators and welders were tested only Friday, while during year 2 AM operators and controls were tested on both Monday and Friday. Lung function was assessed using a hand held PC-based spirometry flow sensor with bi-directional ultrasound transit time analysis (Spirare, Diagnostica, Oslo, Norway), according to guidelines from the American Thoracic Society [30]. Obtained spirometry values in the form of a percentage of predicted forced vital capacity (FVC), forced expiratory volume in one second (FEV1) as well as their ratio (FEV1/FVC) were compared against sex-specific Swedish reference materials [31,32]. Participants’ height and weight were measured before the spirometry using a measuring tape mounted on a wall and a mechanical weight scale, respectively.

### Statistical analyses

Differences in sex distribution and reported indoor air quality problems/symptoms were investigated by chi-square test in Statistica 13 (Dell). Comparisons between groups of workers were performed with t-test. In individuals with values for both Monday and Friday, paired T-test was performed. Continuous variables were found not normally distributed and were therefore log-transformed before analysis. For α1-microglobulin, values below the limit of detection (LOD, 5.1 mg/L) were imputed with LOD/√2 (3.6 mg/L), similar to what has been described for α1-microglobulin [33]. Spearman correlation analysis were done between blood metal levels measured in the present study and urine metal levels that was measured as part of previous published study [7].

An orthogonal partial least squares-discriminant analysis (OPLS-DA) model was used to investigate difference between AM operators and controls regarding all the exposure and clinical markers. Variables important for the separation of the groups were selected using a variable influence on projection (VIP) value ≥ 1.0 and VIP value > standard error (SE). These variables were subsequently included in a second OPLS-DA model with one predictive and one orthogonal component. Model quality was evaluated using R^2^ and Q^2^ describing the goodness of fit and prediction, respectively, and a CV-ANOVA.

## Results

### Participants

All operators working at the AM facility at year 1 were invited to participate in the study, however, at year 2, the number of AM operators was restricted to 12 volunteers. No statistical differences between controls and AM operators could be found regarding sex, age, or BMI. The welders had a significantly larger ratio of males, and also had a higher BMI compared to controls (Table 1).

### Surveys

**Fig 1:**
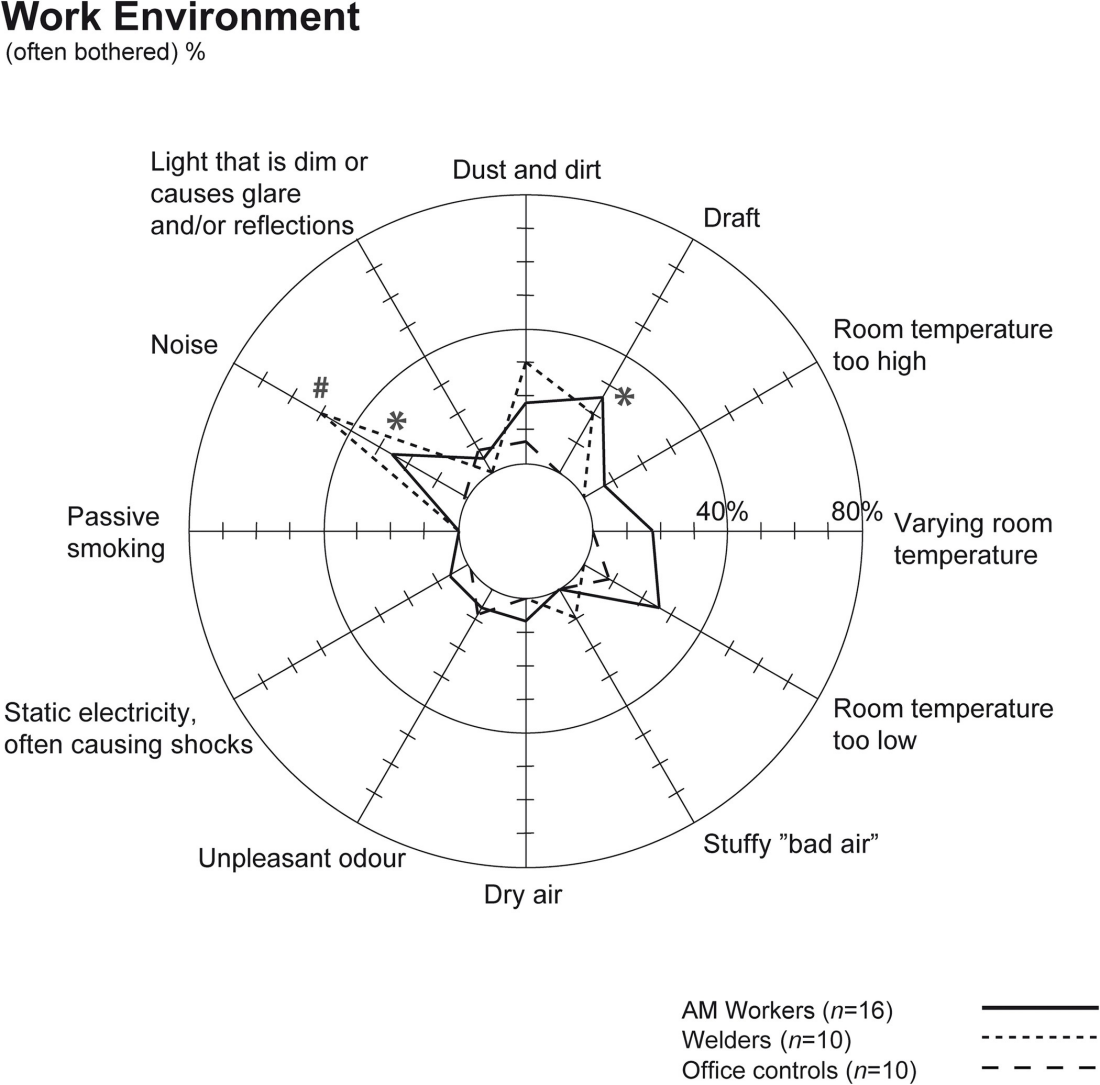
Summary of perceived indoor climate reported from work environment questionnaire, MM040NA. The diagram shows the prevalence of factors reported often (at least once a week) during the last 3 months. * p<0,05 AM vs Control, # p<0.05 Welder vs Control Chi-Square test.

The AM operators reported significantly more frequent (p<0.05) that they were often bothered (that they had a negative experience with a factor at least once per week) with draft/air movement and noise than the controls during the last three months (Fig 1). All participants that experienced problems with draft/air movement were enrolled in year 2, and a statistical comparison showed that AM operators enrolled year 2 had significantly more problems than those enrolled year 1 (p<0.05). There was no statistical difference between AM operators year 1 and 2 for noise. The welders reported significantly more frequent issues (p<0.05) with noise than the controls during the last three months (Fig 1).

The question regarding “often occurring symptoms” (at least once per week) during the last 3 months showed that there were no statistical significances in reported symptoms between AM, welders, and controls (see supplemental S1 Fig). Approximately four times higher prevalence of skin signs/symptoms on the hands including dryness, itching and skin redness (erythema) in both AM operators as well as welders was found, compared to the control group.

### Blood metals

In the current study, metals in blood were investigated in samples from the same individuals collected at the same time point compared to our previous published paper showing levels of metals in urine [8]. Both controls and AM operators showed significantly higher levels of chromium in year 2 when compared to year 1 (Table 2). AM operators had significantly lower nickel levels on Monday year 2 compared to Monday year 1. The welders showed a significant reduction of nickel Friday vs Monday year 1 (Table 2).

**Table 2:** Blood metals.

Group	Year	Day	n	Cr (nmol/L)	Co (nmol/L)	Ni (nmol/L)
**Control**	1	Mon.	10	6.9 (6.1–8.6) ¤¤¤	1.4 (0.9–3.5)	7.5 (4.3–13.9)
	2	Mon.	8	10.1 (8.2–11.9) ¤¤¤	1.0 (0.9–1.3)	5.5 (4.0–9.3)
		Fri.	7	10.3 (9.5–11.7)	1.1 (0.8–1.6)	5.3 (3.0–7.3)
**AM-operators**	1	Mon.	7	7.4 (4.7–16.9) ¤	1.7 (0.8–4.8)	10.8 (6.2–32.0) ¤
		Fri.	6	7.4 (5.4–10.4) ¤	2.1 (0.9–7.7)	8.9 (4.6–16.9)
	2	Mon.	11	10.6 (9.3–12.8) ¤	1.2 (0.6–2.5)	6.2 (4.7–9.8) ¤
		Fri.	8	10.5 (7.1–15.3) ¤	1.4 (0.8–2.3)	6.4 (3.7–10.7)
**Welders**	1	Mon.	10	7.6 (5.4–10.4) **	1.1 (0.8–1.3)	11.4 (7.2–14.5) **
		Fri.	8	7.3 (4.0–10.5) ##	1.1 (0.9–1.4)	7.7 (4.5–10.6) ##

AM–additive manufacturing. Values are the geometric mean (min-max).

.¤ < 0.05, ¤¤¤ < 0.001, Year 2 vs Year 1 same day and group

**p < 0.01, Welders vs Controls same day and year

## Welders Friday vs Monday same year.

**Fig 2:**
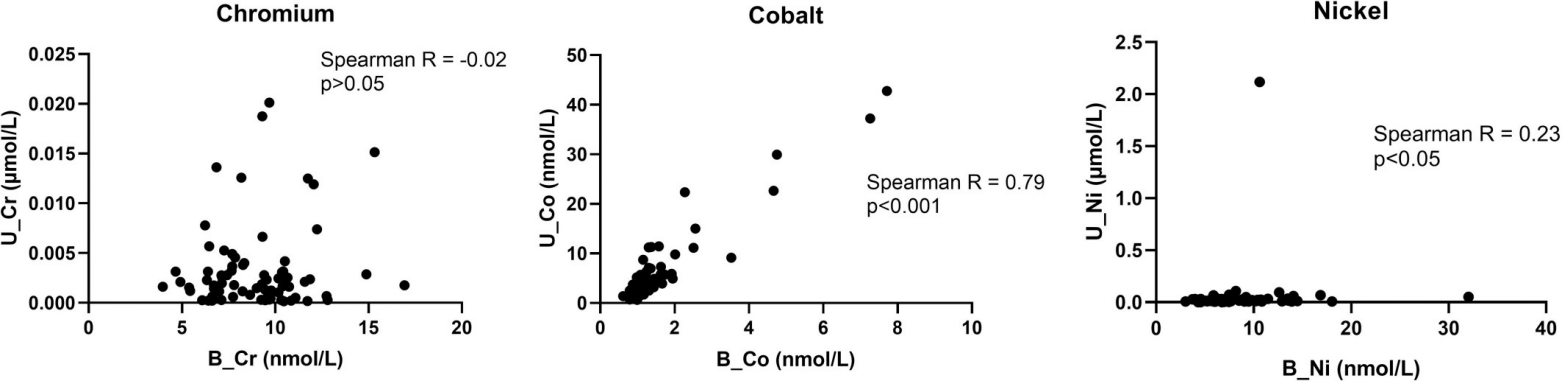
Correlation analysis of cobalt, nickel and chromium in blood and urine.

To investigate a potential relationship between the abundance of metals in urine [8] and in the blood, correlation analyses were performed. Cobalt and nickel showed a significant positive correlation between blood and urine metal levels (Spearman R = 0.79, p<0.001 and R = 0.23, p<0.05 respectively, Fig 2). The correlation for nickel was in part driven by one welder with high levels in urine (>2 μmol/L), when removing this individual as an outlier, the correlation became non-significant (R = 0.21, p = 0.07, see supplemental S2 Fig).

### Clinical analyses

Regarding hepatic and renal function, a reduction of ALP in AM operators was the only significant difference found when comparing Monday and Friday year 2, (Table 3). For the AM operators, there were also non-significant (12–29%) reductions of liver enzymes: ASAT, ALAT, ASAT/ALAT and ALP comparing year 1 and year 2. For the renal function marker α1-microglobulin, there was a non-significant trend that the AM operators had higher levels year 1 compared to year 2 and an increase during the workweek year 1, while during year 2 the values decreased over the workweek (Table 3).

**Table 3:** Clinical analyses of hepatic and renal function.

Group	Year	Day	n	ASAT (μkat/L)	ALAT (μkat/L)	ASAT/ALAT (μkat/μkat)	ALP (μkat/L)	α1-microglobulin (mg/L)
**Reference values**				**<0.75**	**<1.2**		**<1.9**	**<10**
**Control**	1	Mon.	11	0,36 (0,25–0,51)	0,31 (0,18–0,53)	1,19 (0,84–2,00)	0,92 (0,58–1,50)	5,45 (3,60–22,00) [4]
	2	Mon.	8	0,38 (0,25–0,58)	0,35 (0,16–0,71)	1,09 (0,73–1,69)	0,88 (0,63–1,23)	4,06 (3,60–5,49) [2]
		Fri.	7	0,39 (0,25–0,52)	0,35 (0,16–0,71)	1,10 (0,73–1,56)	0,93 (0,70–1,17)	5,03 (3,60–10,70) [3]
**AM-operators**	1	Mon.	7	0,59 (0,34–2,80)	0,51 (0,24–1,80)	1,18 (0,65–1,56)	1,20 (0,80–5,20)	8,94 (3,60–38,00) [5]
		Fri.	6	0,60 (0,28–3,90)	0,51 (0,24–2,20)	1,18 (0,91–1,77)	1,19 (0,75–6,00)	13,34 (3,60–110) † [4]
	2	Mon.	11	0,46 (0,31–0,83)	0,45 (0,19–1,50)	1,02 (0,55–1,89)	0,95 (0,57–1,53) #	8,19 (3,60–31,5) [5]
		Fri.	8	0,45 (0,33–0,70)	0,44 (0,23–1,23)	1,04 (0,51–1,78)	0,85 (0,54–1,27) #	5,58 (3,60–12,10) [5]
**Welders**	1	Mon.	10	0,47 (0,34–1,30)	0,44 (0,27–1,90)	1,06 (0,68–1,47)	1,07 (0,61–2,00)	6,40 (3,60–27,00) [5]
		Fri.	8	0,38 (0,30–0,46)	0,34 (0,27–0,44)	1,12 (0,86–1,41)	1,04 (0,80–1,30)	4,52 (3,60–11,00) †[2]

AM–additive manufacturing; ASAT–Aspartate transaminase; ALAT–alanine transaminase; ALP–alkaline phosphatase. Values are the geometric mean (min-max). Monday and Friday

# p < 0.05, paired t-test in individuals participating both Monday and Friday

† = p<0.05, t-test Welders vs AM same year and day. Reference values were obtained from the Clinical Chemistry section of Linköping University Hospital at the time of analysis. Values within square brackets [] for α1-microglobulin are the number of individuals with values>LOD.

For the lipoproteins (apo B, apo A-I and apo B/apo A-I ratio), the only significant difference was found among controls, where a significantly higher apoB/apoAI ratio was found Monday year 2 compared to Monday year 1 (Table 4). The AM operators showed a weak, non-significant, trend for decreasing apo A-I and increasing apo B/apo A-I ratio during the workweek for both years 1 and 2.

**Table 4:** Plasma apoAI, apo B and apoB/A-I ratio.

Group	Year	Day	n	Apo A-I (g/L)	Apo B (g/L)	ApoB/Apo A-1
**Control**	1	Mon.	11	1.55 (1.29–1.95)	0.82 (0.56–1.09)	0.53 (0.43–0.65) ¤
	2	Mon.	8	1.55 (1.13–2.28)	0.97 (0.65–1.28)	0.63 (0.47–0.84) ¤
		Fri.	7	1.49 (1.07–1.73)	0.90 (0.67–1.23)	0.61 (0.40–0.84)
**AM-operators**	1	Mon.	7	1.47 (1.22–1.65)	0.89 (0.58–1.72)	0.61 (0.38–1.09)
		Fri.	6	1.41 (1.22–1.76)	0.95 (0.65–1.69)	0.67 (0.40–1.23)
	2	Mon.	11	1.54 (1.25–1.94)	0.93 (0.69–1.41)	0.60 (0.42–0.92)
		Fri.	8	1.50 (1.21–1.64)	1.03 (0.71–1.39)	0.69 (0.47–0.85)
**Welders**	1	Mon.	10	1.33 (0.93–1.72)	0.86 (0.56–1.18)	0.65 (0.33–0.84)
		Fri.	8	1.30 (1.08–1.69)	0.83 (0.54–1.02)	0.64 (0.32–0.86)

AM–additive manufacturing; Apo A-I–Apolipoprotein A-I; Apo B–Apolipoprotein B. Values are the geometric mean (min.-max.).

¤ < 0.05, Year 2 vs Year 1 same day and group.

For the antioxidant activity (PON1) and degree of inflammation analysis (SAA1), AM operators participating both Monday and Friday, showed a significant reduction of the SAA1/PON1 ratio over the workweek year 2 (Table 5). Welders participating on both Monday and Friday showed a significant reduction of SAA1 during the workweek year 1 (Table 5). Welders had significantly lower levels of SAA1 compared to AM operators comparing the same day and year.

**Table 5:** Plasma antioxidant activity and degree of inflammation.

Group	Year	Day	n	PON1 AREase (U/mL)	SAA1 (ug/mL)	SAA1/PON1 AREase (μg/U)
**Control**	1	Mon.	11	78 (53–97)	1062 (435–1795)	13.6 (7.23–22.4)
	2	Mon.	8	91 (54–188)	1390 (807–3449)	15.3 (5.5–37.5)
		Fri.	7	79 (56–136)	1121 (617–1687)	14.2 (9.5–27.2)
**AM-operators**	1	Mon.	7	81 (65–95)	1827 (460–7230) †	22.5 (6.4–77.2)
		Fri.	6	80 (60–92)	1607 (304–14759)	20.1 (4.0–179.3)
	2	Mon.	11	90 (65–137)	2062 (1008–4717)	22.9 (10.4–69.4) #
		Fri.	8	84 (58–129)	1387 (617–2455)	16.1 (7.3–29.9) #
**Welders**	1	Mon.	10	77 (52–102)	827 (447–2124) # †	10.9 (4.5–35.3)
		Fri.	8	76 (57–96)	590 (353–1072) #	7.4 (3.7–13.8)

AM–additive manufacturing; PON1 AREase–paraoxanase-1 arylesterase; SAA1 –serum amyloid A1.

Values are the geometric mean (min.-max.).

# p < 0.05, paired t-test in individuals participating both Monday and Friday

† = p<0.05 Welders vs AM same year and day.

### Multivariate model of exposure and clinical markers

**Fig 3:**
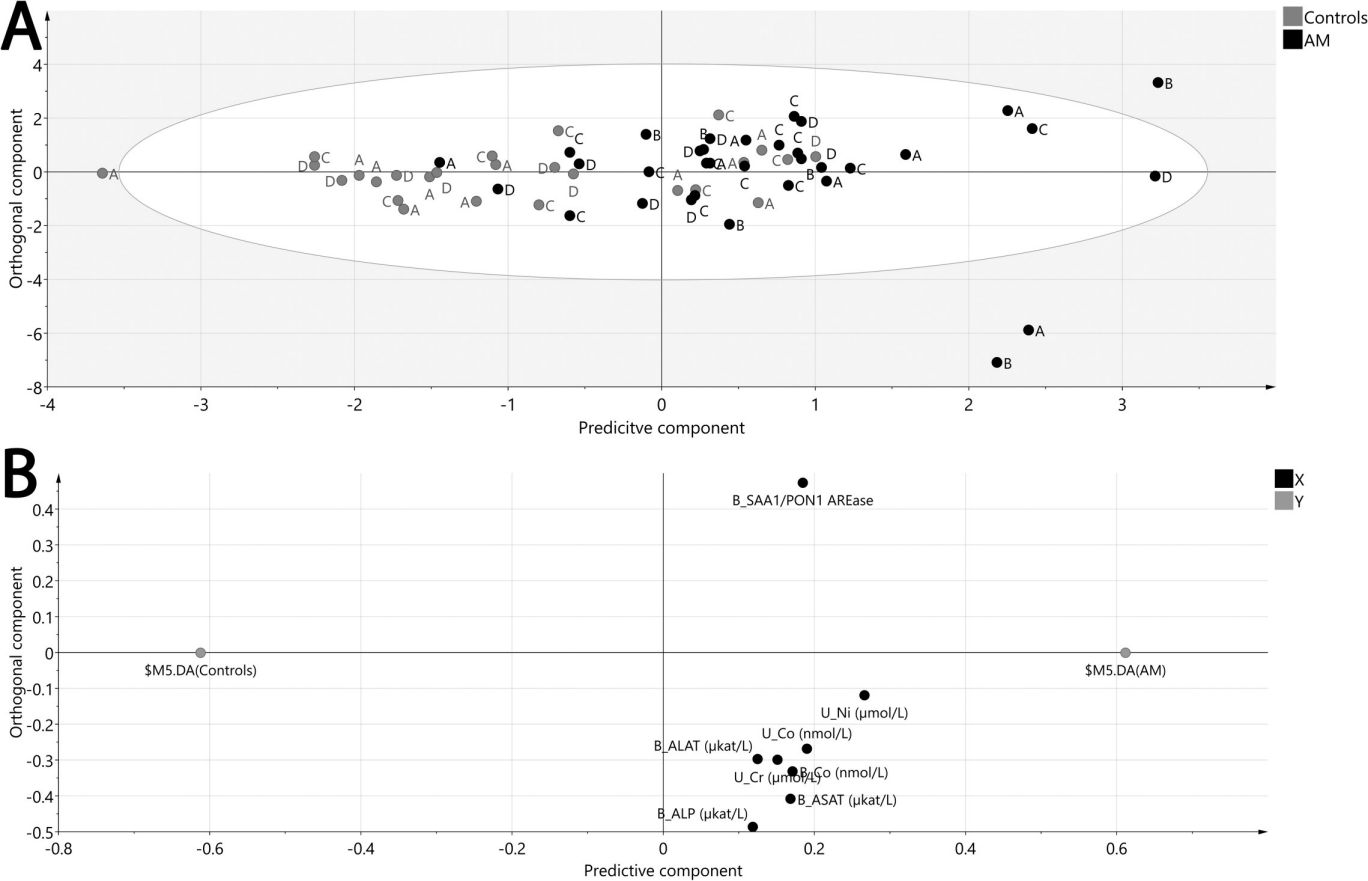
Multivariate modelling using OPLS-DA of AM operators and controls Monday and Friday both years. A. Score plot showing the separation of AM operators (black) and controls (grey) during Monday year 1 (A), Friday Year 1 (B), Monday Year 2 (C) and Friday Year 2 (D). B. Loading plot showing the variables responsible for the separation of samples. R^2^ = 0.34, Q^2^ = 0.30, CV-ANOVA p-value < 0.001.

With the purpose to investigate what separates the AM operators and the controls, including all exposure markers and clinical markers during both years, a multivariate OPLS-DA model was used. This analysis represents a supervised model aiming to find what factors separate the groups, in this case AM operators and controls. The model showed a significant (CV-ANOVA p<0.001), although a weak separation of the AM operators and controls (Fig 3A). This was reflected in a modest goodness of fit (R^2^ = 0.34) and predictability of the model (Q^2^ = 0.30). It did however reveal that eight markers, including SAA1/PON1 AREase, urinary nickel, cobalt and chromium, blood cobalt and the hepatic markers ASAT, ALAT and ALP were higher in the AM operators compared to the controls (Fig 3B).

### Spirometry

AM operators showed a significantly higher FEV1 when comparing Friday year 2 vs year 1 (Table 6). There were no changes comparing Monday and Friday in controls or AM-operators. Approximately 30% of the AM operators deviated from what is considered as normal in the reference interval Friday year 1 (80–120%, Hedenström reference material).

**Table 6:** Spirometry results.

Group	Year	Day	n	FVC (% predicted)	FEV1 (% predicted)
**Reference value**				**>80**	**>80**
**Control**	1	Mon.	0	-	-
	2	Mon.	8	95 (87–110)	96 (80–115)
		Fri.	7	88 (80–96)	88 (82–97)
**AM-operators**	1	Mon.	0	-	-
		Fri.	6	81 (66–94)	80 (68–97) ¤ †
	2	Mon.	12	92 (78–107)	93 (75–109)
		Fri.	9	92 (82–105)	92 (80–106) ¤
**Welders**	1	Mon.	0	-	-
		Fri.	8	92 (85–106)	94 (82–113) †

AM–additive manufacturing; FVC–forced vital capacity, FEV1 –forced expiratory volume in 1 second.

Values ae the geometric mean (min-max) of the expected value for the individual based on age, gender and height with the reference material Hedenström.

¤ < 0.05, Year 2 vs Year 1 same day and group.

† = p<0.05 Welders vs AM same year and day.

## Discussion

As with all emerging technological advances, AM may involve new occupational exposure related health risks that need to be fully addressed. This study focuses upon possible clinical markers for monitoring health effects among AM operators, investigated in a manufacturing plant previously described [8].

### Exposure markers in urine and blood

The main exposure routes in metal working environments are via inhalation, ingestion, or skin contact, where inhalation is the dominating source [34]. To investigate if the body of the exposed individuals has absorbed metal particles from ambient air, metal analyses in blood and urine are performed routinely all over the world. Unfortunately, regulatory action limits for many metals in blood or urine are still missing.

Here, nickel in the AM operators blood showed a significant reduction when comparing year 1 and year 2 (Table 2). This is well in line with findings from our previous study [8], where a (non-significant) reduction of nickel in urine was found. Blood cobalt also showed a similar decreasing trend. The observed decreases of metals in urine and blood at the follow-up are likely due to the improved PPE recommendations and work routine guidelines that the company had implemented. Especially since the used powder remained the same while the number of machines and general production, and powder use, had increased approximately 30% between the years [8]. Interestingly, a non-significant increase of chromium, which is also a component in the studied powder, was found in the AM operator`s blood comparing years 1 and 2. Chromium was also higher in the controls during the same period, indicating either a widespread increase of chromium in the work environment (note that AM operators and controls share the same building and break rooms) or a non-AM related contamination source (industrial activities nearby). One contamination case, unintentionally revealed in the study, was significantly increased urinary manganese in almost all subjects at the time of sampling year 2, compared to year 1 (see supplemental S1 Table), that could not be explained by monitored work-related exposures and after extensive investigations the source turned out to be a temporary external factor.

Welders showed significantly higher levels of nickel in their blood on Monday year 1 compared to controls (Table 2). This finding is most probably explained by welding activities that day or the week before but may also be explained by hobby activities involving nickel exposure during the weekend. The significant decrease of blood nickel during the workweek year 1 may be explained by low work activities in stainless steel materials during the workweek.

Since metals in both whole blood and urine have been measured in the participating individuals, the question whether blood or urine is most suitable for biomonitoring of occupational exposures became obvious. It is definitely less invasive to perform the analyses in urine, but it is possible that some of the metals in the AM powders may not be cleared via urine. To explore this further, the correlation between urine metals published in the previous study [8] and blood metals performed in the current study was investigated, as they were sampled at the same time in the same individuals. Cobalt and nickel showed significant positive correlations although only cobalt showed a strong correlation (Fig 2). Furthermore, there was one welder with high nickel in urine (>2 μmol/L) that seemed to be driving the correlation. When removing this individual as a potential outlier, the correlation coefficient slightly dropped but also become non-significant indicating that only cobalt appears to have a good correlation between blood and urine. The multivariate model indicated that all three metals in urine were associated with the AM operators compared to controls while in blood only cobalt showed a similar association. Since the Finnish Institute of Occupational Health [35] has recommended action levels for these metals in urine and since urine analysis is less invasive, it seem to be the best choice for future studies of metal exposures.

### Clinical markers and health effects

The present study aim to meet the increasing interest in relevant clinical analyses for metal workers, which provides a more comprehensive picture of the worker`s health. However, it is important to remember when studying these results that deviating clinical marker values, one by one, may be explained by several different conditions not related to metal exposure. These results need interpretation with caution and has to be evaluated in larger cohorts before any conclusions could be drawn.

#### Lung function

In the current study, at year 1, approximately 30% of the AM operators got to perform a follow up on the spirometry due to values deviating from the reference interval (of 80–120% of expected values). Since this company had just started their AM production, this finding cannot be explained by AM activities but may be explained by many years in the metal working industry prior to their AM positions. However, the AM operators showed a significant improvement when comparing FEV1 Friday at year 1 and year 2 (Table 6), which is in line with improvements in their work environment. However, until knowledge has improved regarding health risks for AM manufacturing it might be suitable to perform spirometry for new employees and then perform regular follow-ups annually or every 2 years.

#### Vascular function

Since metal exposure may result in detectable metals in blood and urine, it is highly important to study metal related health effects in the vascular system. Studies of plasma protein-nanoparticle interactions have shown that the HDL protein apo A-I is able to interact with metal particles in plasma [23]. Therefore, apo A-I analysis was included in this study (Table 4). Apo A-I/HDL is mainly known to mediate cholesterol efflux via the SR-BI receptor [36] but is also known to have crucial functions in the immune system [21]. The slight, non-significant, decrease in apo A-I found after a work week in AM operators and welders but not controls (Table 4) may result from immunity related activities and is important to follow up in larger studies since decreased apo A-I levels is a known risk factor for cardiovascular complications [37]. The acute phase protein Serum Amyloid A1 (SAA1) is located on HDL-particles and was included as a marker of acute inflammatory response. No significant increase was found in this study, but interestingly AM operators had in general higher levels of SAA1 than welders and controls (Table 5). A recent review concluded that even small increases of SAA1 levels are associated with an increased risk of cardiovascular disease and that such increases have been shown for other occupational exposures [38]. Thus, the potential increase of SAA1 due to metal AM should be further investigated in larger studies. It would also be realistic to expect oxidative stress in the circulation as result of metal exposure. Therefore, the antioxidant activity of PON1 were investigated. This showed a slight, non-significant, decrease in activity in AM operators comparing Monday and Friday at year 2 (Table 5) but surprisingly less year 1, when the exposure was more pronounced. The SAA1/PON1 ratio is being used as a vascular status marker [24] and the multivariate model indicated that this marker was associated with the AM operators in comparison with controls. However, it is not clear whether the SAA1/PON1 ratio is a useful tool investigating metal exposure related health effects, so this has to be further studied.

#### Hepatic function

Based on previous findings, metal particles may be cleared via faeces, and thereby may affect hepatic function. It has been shown that 35% of cobalt oxide particles (1.4 and 2.7 μm) were cleared via faeces in an inhalation study of rats [39]. This scenario is highly relevant for AM environments. In line, negative effects on liver function such as oxidative stress as result of exposure to cobalt has been previously described [40] and chromium, has been shown to have hepatotoxic effects [16]. In the present study, some AM operators did have ASAT and ALP values that exceeded the upper reference limit (>0.75 μkat/L for ASAT and >1.9 μkat/L for ALP) at year 1, when the exposure had been most pronounced, and specifically after a workweek (Table 3). Interestingly, the welders did not show these values, possibly since their exposure is mainly nanoparticles [8] that may be cleared to a higher extent via urine. Furthermore, all three hepatic markers showed an association to the AM operators in the OPLS-DA model indicating that these may be of interest for future research regarding their usefulness for metal exposure health assessment.

#### Renal function

Since biological uptake of metals is routinely studied by analysis of urine, it could be expected that renal function may be affected. A risk of negative effects on renal function as result of chromium exposure [16] has recently been suggested. In this study, the clinical marker of renal function α1-microglobulin showed a non-significant trend for higher values in AM operators compared to controls (Table 3) and specifically after a workweek at year 1, which is consistent with the higher levels of airborne particles in the work environment that year. Regarding the welders, some individuals exceeded the upper reference value (>10 mg/L) on Monday, which is consistent with the increased exposure in welders described above. Since welding activities involve higher levels of nanoparticles, the possibility of using α1-microglobulin as a clinical marker of renal function has to be further investigated.

### Survey

A general survey of the work environment done as a screening whether the participants experienced occupational-related problems with the building or had symptoms they connected to work. The survey showed that the AM operators experienced no significant symptoms that they related to their workplace as compared to the controls, although the both AM operators and welders had an approximate four-fold higher incidence of skin signs/symptoms on the hands than controls (see supplemental S1 File). This might be due to their handling of metallic components, although it could also be related to their manual labor and use of gloves in their work. The survey also includes questions regarding factors in the work environment. These indicated that AM operators were significantly more bothered with draft/air movement as well as noise compared to the controls. The draft/air movement may be related to both the ventilation system as well as a possibility that the gates of the industrial building were opened to allow transportations. All the participants that experienced issues with draft/air movement were enrolled year 2 and were newly hired. As such, it is possible that they noticed problems that more experienced individuals took for granted. The experienced problems with noise are not surprising due to the industrial activity.

### Limitations

A major limitation of this study is the limited number of study participants. However, as this is one of the world’s first metal AM serial production plants, it gives an insight into what potential occupation health hazards that might need to be addressed and provides a basis for future research. The α1-microglobulin measurement have a high LOD compared to the reference value (5.1 vs 10 mg/L), and many measures were below LOD. These were imputed using LOD/√2, which is a rather simplistic approach that could introduce bias. However, the obtained imputation value of 3.6 mg/L were relatively similar to normal values reported in other studies such as 2.4 mg/L [41] as well as 4.2 mg/L [18], thus strengthening the use of the simplistic imputed value.

## Conclusions

The current study indicates that despite earlier published differences in airborne exposures and operating procedures comparing AM operators and welders, the performed clinical analyses seems relevant for both metal professions. Results suggest that the clinical markers for renal, hepatic, and vascular function may be a useful complement to exposure marker analyses, such as urine or blood metals, and traditional lung function analyses for a more complete picture of metal AM worker`s health. However, validation in larger cohorts is necessary before any conclusions could be drawn.

## Supporting information

S1 Fig Frequent symptoms from the occupational environment obtained by questionnaire. (DOCX)

S2 Fig Spearman correlation analysis of nickel in urine and blood after removal of potential outlier. (DOCX)

S1 Table Circulating blood metal values. (DOCX)

S1 File Survey instrument. (PDF)
