# Supplementary material for: Metal additive manufacturing and possible clinical markers for the monitoring of exposure-related health effects
Source: PLoS One. 2021 Mar 18;16(3):e0248601. doi: 10.1371/journal.pone.0248601 (PMC7971853; doi:10.1371/journal.pone.0248601)
Supplement: S1 Fig — (DOCX) [file pone.0248601.s001.docx]

**S1 Figure**. Frequent symptoms from the occupational environment obtained by questionnaire.


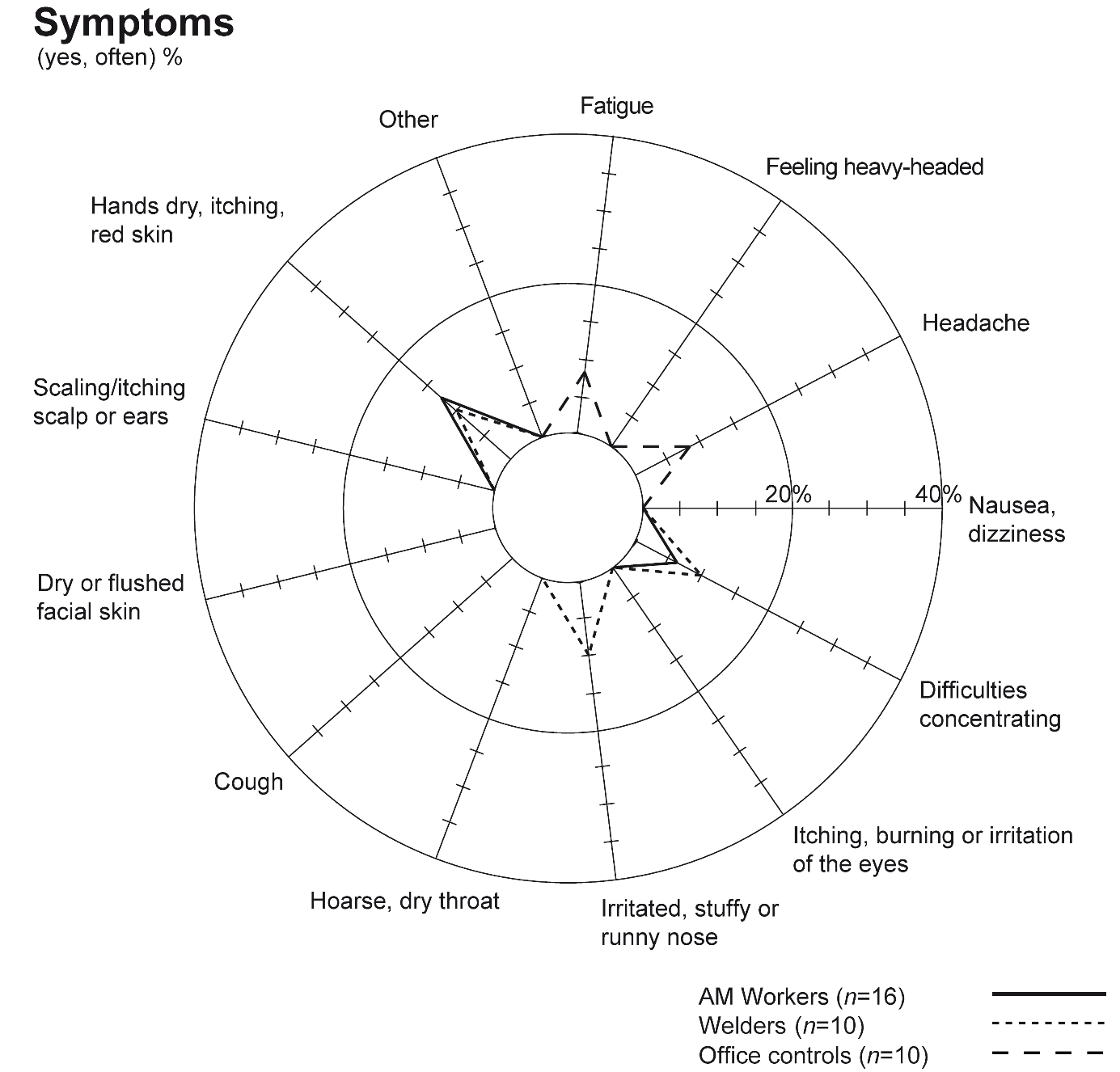


**S1 Figure**. Summary of perceived symptoms from work environment questionnaire, MM040NA. The diagram shows the prevalence of symptoms reported often (at least once a week) during the last 3 months. * p<0,05 AM vs Control, # p<0.05 Welder vs Control Chi-Square test.
