# Supplementary material for: Metal additive manufacturing and possible clinical markers for the monitoring of exposure-related health effects
Source: PLoS One. 2021 Mar 18;16(3):e0248601. doi: 10.1371/journal.pone.0248601 (PMC7971853; doi:10.1371/journal.pone.0248601)
Supplement: S2 Fig — (DOCX) [file pone.0248601.s002.docx]

**S2 Figure**. Correlation analysis of nickel in blood and urine after removing a welder with high nickel in urine as a potential outlier.


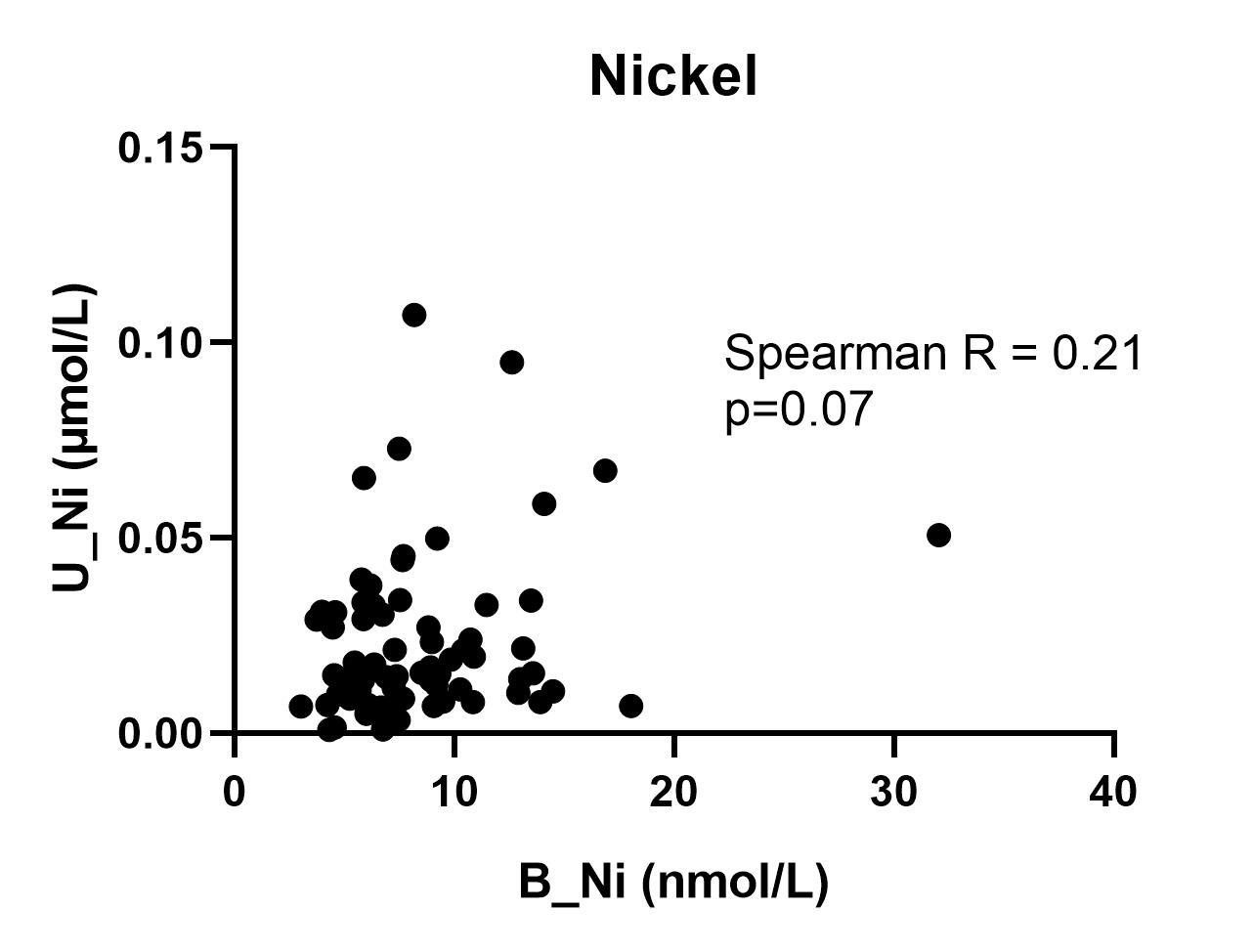


**S2 Figure.** Spearman correlation analysis of nickel in blood and urine after removal of potential outlier.
