# Supplementary material for: Metal additive manufacturing and possible clinical markers for the monitoring of exposure-related health effects
Source: PLoS One. 2021 Mar 18;16(3):e0248601. doi: 10.1371/journal.pone.0248601 (PMC7971853; doi:10.1371/journal.pone.0248601)
Supplement: S1 Table — (DOCX) [file pone.0248601.s003.docx]

**S1 Table:** Circulating blood metal values.

| **Group** | **Year** | **Day** | **n** | **Cr (nmol/L)** | **Mn (nmol/L)** | **Co (nmol/L)** | **Ni (nmol/L)** | **Cd (nmol/L)** | **Sb (nmol/L)** | **Pb (nmol/L)** |
| --- | --- | --- | --- | --- | --- | --- | --- | --- | --- | --- |
| Control | Year 1 | Mon. | 10 | 6,9 (6,1-8,6) | 151,5 (109,2-247,1) | 1,4 (0,9-3,5) | 7,5 (4,3-13,9) | 1,3 (0,6-4,2) | 17,1 (15,6-19,3) | 52,8 (29,1-96,5) |
|  | Year 2 | Mon. | 8 | 10,1 (8,2-11,9) | 141,3 (117,6-171,6) | 1,0 (0,9-1,3) | 5,5 (4,0-9,3) | 1,1 (0,8-1,5) | 14,4 (11,7-19,2) | 48,3 (25,0-66,4) |
|  |  | Fri. | 7 | 10,3 (9,5-11,7) | 159,6 (121,8-206,4) | 1,1 (0,8-1,6) | 5,3 (3,0-7,3) | 1,3 (0,8-1,8) | 12,9 (9,9-18,2) | 50,4 (30,7-68,9) |
| AM-operators | Year 1 | Mon. | 7 | 7,4 (4,7-16,9) | 146,9 (67,7-233,9) | 1,7 (0,8-4,8) | 10,8 (6,2-32,0) | 1,1 (0,6-2,0) | 17,7 (14,3-31,5) | 53,3 (20,6-388,6) |
|  |  | Fri. | 6 | 7,4 (5,4-10,4) | 139,4 (114,6-158,3) | 2,1 (0,9-7,7) | 8,9 (4,6-16,9) | 1,1 (0,7-1,9) | 18,1 (17,0-20,1) | 51,7 (25,9-157,8) |
|  | Year 2 | Mon. | 11 | 10,6 (9,3-12,8) | 163,1 (103,5-240,6) | 1,2 (0,6-2,5) | 6,2 (4,7-9,8) | 1,4 (0,7-7,3) | 13,6 (11,0-16,8) | 46,4 (24,8-92,6) |
|  |  | Fri. | 8 | 10,5 (7,1-15,3) | 151,6 (86,0-261,0) | 1,4 (0,8-2,3) | 6,4 (3,7-10,7) | 1,5 (0,7-7,6) | 12,9 (10,5-17,4) | 45,0 (17,0-95,5) |
| Welders | Year 1 | Mon. | 11 | 7,6 (5,4-10,4) | 136,7 (91,6-188,5) | 1,1 (0,8-1,3) | 11,3 (7,2-14,5) | 2,3 (0,8-9,6) | 16,0 (12,7-20,5) | 48,0 (22,2-88,8) |
|  |  | Fri. | 7 | 7,2 (4,0-10,5) | 130,1 (88,5-254,9) | 1,1 (0,9-1,4) | 7,3 (4,5-10,3) | 2,8 (0,7-10,2) | 17,0 (14,5-19,7) | 43,7 (28,4-90,5) |

Values ae the geometric mean (min-max).
