# Supplementary material for: Metal additive manufacturing and possible clinical markers for the monitoring of exposure-related health effects
Source: PLoS One. 2021 Mar 18;16(3):e0248601. doi: 10.1371/journal.pone.0248601 (PMC7971853; doi:10.1371/journal.pone.0248601)
Supplement: S1 File — (PDF) [file pone.0248601.s004.pdf]

INDOOR CLIMATE<br>Work environment<br>ENGLISH VERSION	MM 040 NA<br>Date<br>year month day<br>|___|___|___|___|___|___|	Name (voluntary information)
1-6		Company/Institution (important information)
7-11 Occupation|___|___|___|___|___|		Department (important information)
12-21 User |___|___|___|___|___| Group |___|___|___|___|___| Filled in by investigator		

This questionnaire concerns your indoor climate and possible symptoms you may be experiencing.

BACKGROUND FACTORS

22-23 Year of birth 19 |___|___|

24 Sex Male ☐1 Female ☐2

25 Do you smoke? Yes ☐1 No ☐2

Occupation ....

How long have you been at your present place of work? |___|___| years 26-27

WORK ENVIRONMENT

Have you been **bothered** during **the last three months** by any of the following factors at **your work place**?
(Answer every question even if you have not been bothered!)

		Yes, often (every week) (1)	Yes, sometimes (2)	No, never (3)
28	Draught	☐	☐	☐
29	Room temperature too high	☐	☐	☐
30	Varying room temperature	☐	☐	☐
31	Room temperature too low	☐	☐	☐
32	Stuffy "bad" air	☐	☐	☐
33	Dry air	☐	☐	☐
34	Unpleasant odour	☐	☐	☐
35	Static electricity, often causing shocks	☐	☐	☐
36	Passive smoking	☐	☐	☐
37	Noise	☐	☐	☐
38	Light that is dim or causes glare and/or reflections	☐	☐	☐
39	Dust and dirt	☐	☐	☐

WORK CONDITIONS

		Yes, often (1)	Yes, sometimes (2)	No, seldom (3)	No, never (4)
40	Do you regard your work as interesting and stimulating?	☐	☐	☐	☐
41	Do you have too much work to do?	☐	☐	☐	☐
42	Do you have any opportunity to influence your workin conditions?	☐	☐	☐	☐
43	Do your fellow-workers help you with problems you may have in your work?	☐	☐	☐	☐

75-80 | | | | | | |

Andersson K. Epidemiological approach to indoor air problems. Indoor Air 1998;suppl 4:32-9.
Dept of Occup and Environm Med, Örebro University Hospital, Sweden. Fax +46 19 120404.
E-mail kjell.andersson@orebroll.se. Version 8910-2 K.Andersson©/IF.
The questionnaire is free to use in research and non-commercial activities.

Please turn ⇨

PAST/PRESENT DISEASES/SYMPTOMS

		Yes (1)	No (2)	If Yes: during the last year? Yes (1)	No (2)
1-2	Have you ever had asthmatic problems?	☐	☐	☐	☐
3-4	Have you ever suffered from hayfever?	☐	☐	☐	☐
5-6	Have you ever suffered from eczema?	☐	☐	☐	☐

PRESENT SYMPTOMS

During the **last three months** have you had any of the following symptoms?
(Answer every question even if you have not had any symptoms!)

		Yes, often (every week) (1)	Yes, sometimes (2)	No, never (3)	**If YES:** do you believe that it is due to your work environment? Yes (1)	No (2)
7-8	Fatigue	☐	☐	☐	☐	☐
9-10	Feeling heavy-headed	☐	☐	☐	☐	☐
11-12	Headache	☐	☐	☐	☐	☐
13-14	Nausea/dizziness	☐	☐	☐	☐	☐
15-16	Difficulties concentrating	☐	☐	☐	☐	☐
17-18	Itching, burning or irritation of the eyes	☐	☐	☐	☐	☐
19-20	Irritated, stuffy or runny nose	☐	☐	☐	☐	☐
21-22	Hoarse, dry throat	☐	☐	☐	☐	☐
23-24	Cough	☐	☐	☐	☐	☐
25-26	Dry or flushed facial skin	☐	☐	☐	☐	☐
27-28	Scaling/itching scalp or ears	☐	☐	☐	☐	☐
29-30	Hands dry, itching, red skin	☐	☐	☐	☐	☐
31-32	Other ...….....................................	☐	☐	☐	☐	☐

FURTHER COMMENTS

....

....

....

....

....

THANK YOU!
